# Current evidence supporting associations of DNA methylation measurements with survivorship burdens in cancer survivors: A scoping review

**DOI:** 10.1002/cam4.7470

**Published:** 2024-07-04

**Authors:** Michael Sayer, Ding Quan Ng, Raymond Chan, Kord Kober, Alexandre Chan

**Affiliations:** ^1^ School of Pharmacy and Pharmaceutical Sciences University of California Irvine Irvine California USA; ^2^ School of Nursing and Health Sciences Flinders University Adelaide South Australia Australia; ^3^ School of Nursing University of California San Francisco San Francisco California USA

**Keywords:** cancer survivorship, differential DNA methylation, epigenetic aging, epigenetics, literature review, multivariate modeling, study design

## Abstract

**Introduction:**

Identifying reliable biomarkers that reflect cancer survivorship symptoms remains a challenge for researchers. DNA methylation (DNAm) measurements reflecting epigenetic changes caused by anti‐cancer therapy may provide needed insights. Given lack of consensus describing utilization of DNAm data to predict survivorship issues, a review evaluating the current landscape is warranted.

**Objective:**

Provide an overview of current studies examining associations of DNAm with survivorship burdens in cancer survivors.

**Methods:**

A literature review was conducted including studies if they focused on cohorts of cancer survivors, utilized peripheral blood cell DNAm data, and evaluated the associations of DNAm and survivorship issues.

**Results:**

A total of 22 studies were identified, with majority focused on breast (*n* = 7) or childhood cancer (*n* = 9) survivors, and half studies included less than 100 patients (*n* = 11). Survivorship issues evaluated included those related to neurocognition (*n* = 5), psychiatric health (*n* = 3), general wellness (*n* = 9), chronic conditions (*n* = 5), and treatment specific toxicities (*n* = 4). Studies evaluated epigenetic age metrics (*n* = 10) and DNAm levels at individual CpG sites or regions (*n* = 12) for their associations with survivorship issues in cancer survivors along with relevant confounding factors. Significant associations of measured DNAm in the peripheral blood samples of cancer survivors and survivorship issues were identified.

**Discussion/Conclusion:**

Studies utilizing epigenetic age metrics and differential methylation analysis demonstrated significant associations of DNAm measurements with survivorship burdens. Associations were observed encompassing diverse survivorship outcomes and timeframes relative to anti‐cancer therapy initiation. These findings underscore the potential of these measurements as useful biomarkers in survivorship care and research.

## 1 INTRODUCTION

While significant advances have improved cancer treatment, patients still cope with persistent survivorship issues. In studies comparing health status of cancer survivors and healthy counterparts, survivors were many times more likely to experience chronic and severe illnesses.^1^, ^2^ Examples range from impaired cognition, increased anxiety and depression, excessive fatigue, and an increased likelihood of developing cardiovascular comorbidities.^3^, ^4^, ^5^, ^6^ These issues likely contribute to a reduced quality of life for cancer survivors, even years removed from active treatment.^7^, ^8^, ^9^, ^10^

There are many speculated reasons for these persistent issues in cancer survivors. Anti‐cancer treatment has persistent side effects due to the damage it causes to healthy tissues.^11^ Physical changes observed during cancer treatment such as reduced musculature and altered hormone composition often remain long after completion of therapy.^12^, ^13^ Coping with the stressors of cancer can also have a lasting negative impact on mental health.^14^ Quantifiable biomarkers describing cancer's impact may more fully describe why survivorship issues occur and allow clinicians to better address and manage them.

Survivorship struggles may be linked to epigenetic changes induced by cancer. Exposure to toxic anticancer therapy agent(s) combined with excessive biologic, physical and emotional stress can trigger epigenetic modifications.^15^, ^16^, ^17^ Changes in DNA methylation (DNAm), adding or removing methyl groups at the 5′‐position of DNA cytosine residues, are epigenetic modifications that can significantly alter gene expression.^18^, ^19^ Thus, methylation changes at key sites can lead to observed phenotypic changes contributing to survivorship issues. Multiple studies have shown patients treated with chemotherapy having lasting alterations in DNAm at specific sites in peripheral blood samples.^20^, ^21^, ^22^ Evaluating differentially methylated CpG sites or regions in cancer survivors with survivorship issues can provide insights into their biological causes.

In addition to DNAm at specific genomic sites, epigenetic age metrics can provide practical insights. These metrics use collections of CpG sites to predict outcomes such as chronological age, mortality risk, and accelerated aging processes.^23^, ^24^, ^25^, ^26^, ^27^, ^28^ Each epigenetic clock is distinct based on the sample populations used to create them, the genomic regions they emphasize, and their outcomes of interest. Increases in epigenetic age, or elevated epigenetic age relative to chronological age, have been associated with poorer health outcomes including the onset of cancer and increased cumulative comorbidity burden.^29^, ^30^ Cancer survivors have shown accelerated aging over time and accelerated aging relative to non‐cancer controls.^31^, ^32^ Given this, epigenetic aging may be predictive of survivorship burdens.

DNAm data may provide needed insights into the underlying causes of survivorship burdens and eventually identify at risk patients. With this review, we collected studies in the literature that examined the association between DNAm data and cancer survivorship burdens. Survivorship burdens were defined as side‐effects or sub‐optimal health outcomes associated with cancer or anti‐cancer therapy. The overarching aim of this scoping review is to provide an overview of current studies examining associations of DNAm with survivorship burdens and provide recommendations to improve future studies.

## 2 METHODS

**FIGURE 1:**
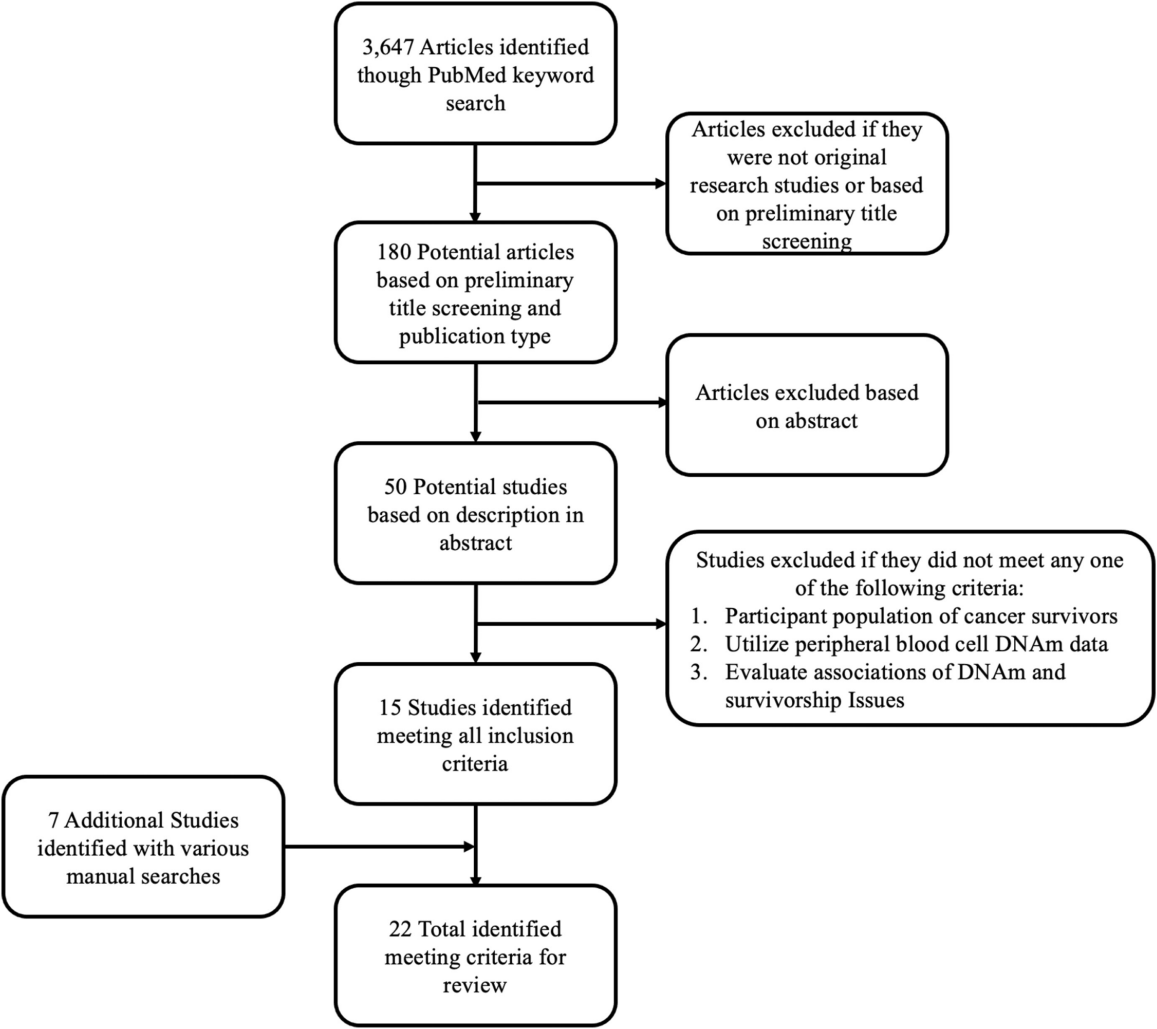
Literature search process. The flowchart describes the process identifying studies for inclusion in this review. First potential studies were identified using keyword searches in PubMed, then studies were excluded based on preliminary title screening and publication type, followed by subsequent abstract screening. Studies were included in the study meeting inclusion criteria both from literature search and manual search techniques.

Literature search was performed using the PubMed search engine from 09/01/23 to 09/06/23, with no restrictions on publication timeframe for potential studies (Figure 1). Combinations of key words and phrases were utilized, which included: cancer, cancer survivor, cancer treatment, chemotherapy, DNA Methylation, differential DNA methylation, epigenetic aging, side effects, survivorship, and quality of life (Table S1). In addition to key word searches, manual searches were performed speaking with researchers in the field and evaluating publications from institutions and research groups associated with cancer survivorship and epigenetics provided additional literature (Figure 1). Inclusion criteria for studies of interest included: (1) Utilization DNA methylation data from peripheral blood samples, (2) Inclusion of cohorts of cancer patients or survivors, and (3) Examination of associations between DNA methylation measurements with survivorship burden(s). After identifying studies through manual and key word searches, they were subsequently utilized in citation searches where studies citing them were evaluated for inclusion.

## 3 DEFINITIONS

### 3.1 Five survivorship burden domains

Specific survivorship burdens assessed were broadly categorized into five domains for organizational purposes based on common reported survivorship issues.^33^, ^34^, ^35^, ^36^, ^37^, ^38^, ^39^ These included Neurocognition, Psychiatry, General wellness, Chronic conditions, and Treatment specific toxicities. Neurocognitive outcomes are reflected in several different categories including executive function, complex attention, language, learning, memory, and motor function. These outcomes are captured by a clinically validated self‐reporting surveys and objective computerized cognitive tests. Psychiatric outcomes reflect the extent of distressing thought, emotional, or behavioral patterns experienced. These are captured with clinical diagnoses of psychiatric disorders such as anxiety or depression, or self‐reported symptoms through clinically validated surveys. General wellness is broadly defined as outcomes related to cancer survivors' quality of life, not reflected in neurocognition or psychiatric domains. This includes general symptoms such as fatigue or pain, evaluations of functional daily living, and social determinants of health such as income status or extent of socialization. Chronic conditions are common comorbidities within the general population that cancer survivors experience many years after cancer treatment, captured with retrospective chart review. Treatment toxicities are specific symptoms or side effects associated with a specific anticancer agent, captured by clinician diagnosis. Domains for neurocognition, psychiatry, general wellness and chronic conditions are attributed to a more general cancer experience and cancer treatments.

### 3.2 DNA methylation measurement timing

In this review, the timing of DNAm measurements was considered relative to the initiation of anticancer therapy within 3 distinct timing windows. Those windows were within 3 months of starting therapy, after 3 months and up to 1 year, and more than 1 year removed from therapy initiation. For studies having protocols not detailing timing of methylation measurements, median time removed for all patients from therapy initiation or active treatment was utilized instead. To provide additional context, we have also described measurement timing relative to completion of anti‐cancer therapy based on described study design. These categories include prior to treatment (for longitudinal studies), during treatment, immediately after treatment, and more than 1 month removed from treatment.

### 3.3 Epigenetic clocks

In this review, we will evaluate epigenetic clocks as a biomarker of aging. This review refers to 7 different epigenetic aging metrics, which will briefly be described here to provide context (Table S3). The Horvath and Hanmum clocks were designed to predict chronological ages, based on the methylation levels of an optimized collection CpG sites.^23^, ^26^, ^28^ Intrinsic epigenetic age is a derivation of Horvath's clock, incorporating immune cell levels as a normalization method accounting for expected changes in cellular composition with aging.^23^ Extrinsic epigenetic age is a derivation of Hannum's clock, using immune cell composition instead to reflect immune specific aging processes.^26^ The predicted outcomes for Levine's (Phenoage) and Grimage are not exclusively chronological age, but are additionally designed to capture mortality risk.^24^, ^27^ For Levine's, a collection CpG sites were selected best capturing mortality risk determined through key serum biomarker levels in common chronic diseases.^27^ Similarly, the GrimAge metric selected CpG sites actually predicting key serum biomarker levels and smoking packyears.^24^ Subsequent transformations of assessed risk incorporating additional data points provide outputs of epigenetic ages utilized. DunedinPace differs from these metrics, as it captures a rate of biological aging. Its optimized collection of CpG sites best predict changes in key biomarkers reflective of aging.^25^ Accelerated aging is captured with DunedinPace values greater than 1, with values less than 1 suggesting a lower aging rate, and a value of 1 being an expected aging rate. Associations of epigenetic aging with various health outcomes aside from age and mortality risk are intuitive, given their associations with aging and their usage of data relevant to various disease etiologies. While changes or differences in methylation status of individual CpG sites or genomic regions can be informative, the intuitive nature of these metrics may make them more impactful for survivorship care applications.

## 4 RESULTS

### 4.1 Search results

Utilization combinations of key words previously described yielded a total of 3647 potential studies on Pub‐Med (Figure 1, Table S1). From these initial studies, 180 were considered based on publication type and title screening (Figure 1). Of these 180 initial studies, 50 were further considered after abstract review (Figure 1). Lastly, review of 50 studies identified 15 that meet outlined inclusion criteria for the review (Figure 1). An additional seven studies were found utilizing various manual searches (Figure 1). In total, 22 studies were discovered meeting inclusion criteria for this review (Table S3).^19^, ^40^, ^41^, ^42^, ^43^, ^44^, ^45^, ^46^, ^47^, ^48^, ^49^, ^50^, ^51^, ^52^, ^53^, ^54^, ^55^, ^56^, ^57^, ^58^, ^59^, ^60^

### 4.2 Patient populations examined

**FIGURE 2:**
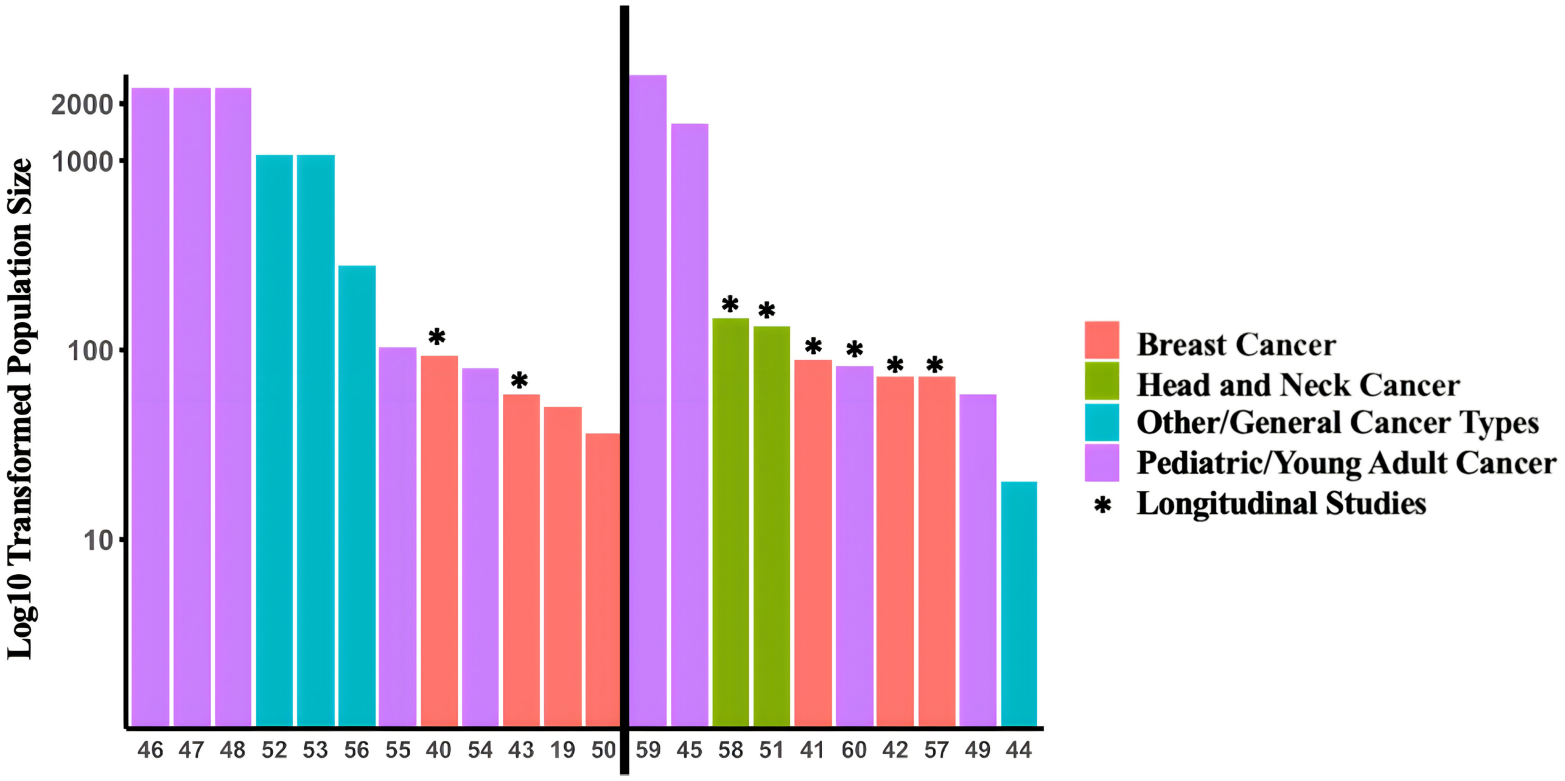
Study population and study design characteristics. The *y*‐axis represents study population size in a log 10 scale, with the *x*‐axis representing study citation number. The coloration of bars represents cancer population type, with asterisks (*) over bars identifying longitudinal studies. Studies to the left of the black line are those utilizing differential methylation analysis while those to the right are utilizing epigenetic aging analysis.

Cancer sub‐types within study populations included: nine (40.8%) childhood/young adult cancer populations,^45^, ^46^, ^47^, ^48^, ^49^, ^54^, ^55^, ^59^, ^60^ seven (31.8%) breast cancer patient populations,^19^, ^40^, ^41^, ^42^, ^43^, ^50^, ^57^ two (9.1%) head and neck cancer populations,^51^, ^58^ and four studies classified as other or non‐specific cancer sub‐types^44^, ^52^, ^53^, ^56^ (Table 1; Figure 2; Table S3). Population sizes ranged from 20 to 2846 cancer patients; there were 11 (50%) studies with cohorts of 100 or less cancer patients^19^, ^40^, ^41^, ^42^, ^43^, ^44^, ^49^, ^50^, ^54^, ^57^, ^60^ (Table 1, Figure 2, Table S3). Control subjects were recruited in 10 total (45.5%) studies, with eight (36.4%) studies recruiting healthy matched controls^40^, ^41^, ^45^, ^46^, ^47^, ^48^, ^49^, ^59^ and two (13.6%) recruiting asymptomatic cancer patients (asymptomatic relative to survivorship burden)^19^, ^55^ (Table 1). With respect to treatment exposures, four (18.2%) studies limited their sample to patients treated with specific chemotherapies,^19^, ^54^, ^55^, ^56^ three (13.6%) limited their samples to patients receiving radiation therapy,^51^, ^54^, ^58^ while most studies did not limit patient populations based on treatment exposures (Table 1).

**TABLE 1:** Study population characteristics and outcomes assessed (n=22).

Category	Count	Percentage
Cancer populations assessed		
Pediatric/young adult cancer	9	40.9
Breast cancer	7	31.8
Head and neck cancer	2	9.1
Other	4	18.2
Population sizes		
Less than 100	11	50.0
Greater than 100	11	50.0
Populations by treatment exposure		
Non‐specific	16	72.7
Specific chemotherapy agent	4	18.2
Radiation therapy	3	13.6
Utilization of controls		
Healthy matched controls	8	36.4
Asymptomatic cancer patients	2	13.6
No controls	12	72.7
Survivorship burdens assessed		
Cognition	5	22.7
Psychiatry	3	13.6
General wellness	9	40.9
Chronic comorbidities	5	22.7
Side effects	4	18.2

*Note*: An overview of various study population characteristics and survivorship outcomes assessed. This includes cancer types, population sizes, treatment exposures, recruitment of controls, and the number of studies assessing outcomes within each survivorship domain. The count column shows the numerical count of studies matching each category described while the percentage column shows the percentage of total studies represented for that category (22 total studies).

### 4.3 Survivorship burdens assessed

**FIGURE 3:**
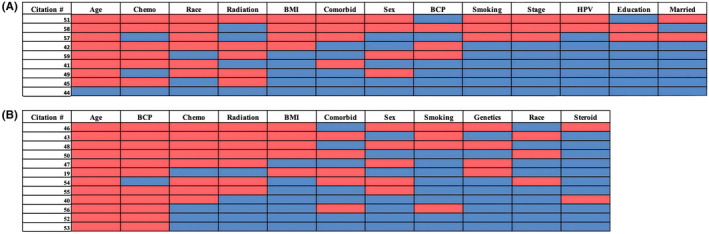
Heatmaps showing covariates utilized in multivariate analysis with methylation data. For panels A and B, column 1 represents the citation number for each row as identified in Table S2. Each subsequent column represents a potential covariate utilized and each row represents a study within the review. Boxes within the heatmap having red coloration indicate the study utilized a covariate, while blue boxes indicate a study did not. Panel A shows covariates utilized in epigenetic age analysis studies, while panel B shows covariates utilized in differential methylation analysis studies. Relevant abbreviations utilized within the figure include: BCP, blood cell proportions; BMI, body mass index; Chemo, chemotherapy received; Comorbid, comorbidities; HPV, human papilloma virus.

Survivorship outcomes were assessed in patients through a combination of objective assessments in seven studies^42^, ^43^, ^44^, ^46^, ^49^, ^54^, ^55^ (31.8%), medical record review for four studies^45^, ^47^, ^59^, ^60^ (18.2%), and patient self‐reported responses in 13 studies (59.1%) studies^19^, ^40^, ^41^, ^44^, ^47^, ^49^, ^50^, ^51^, ^52^, ^53^, ^56^, ^57^, ^58^ (Table 1). Survivorship burdens related to cognition were evaluated in five studies, including outcomes related to self‐perceived cognitive decline evaluated with validated surveys and computerized cognitive assessments evaluating domains like psychomotor speed, learning and memory, attention, processing speed and executive function, and cumulative neurocognitive index.^40^, ^41^, ^42^, ^43^, ^44^ Three studies had survivorship burdens evaluated with psychiatric implications, identifying symptomatic patients with clinically validated surveys and chart review. Outcomes evaluated included depression, anxiety, and cumulative psychiatric symptom burden.^42^, ^44^, ^52^ Survivorship burdens classified as general wellness were assessed in nine studies, with outcomes captured utilizing a variety of objective assessments and survey tools. These outcomes included: pain, fatigue, sleep quality, quality of life, overall physical function or strength, and social determinants of health.^19^, ^41^, ^42^, ^44^, ^48^, ^49^, ^50^, ^51^, ^57^, ^58^ Survivorship burdens related to the development of chronic comorbidities were assessed in five studies and were captured with patient medical record review. Examples of comorbidities included: chronic cardiovascular diseases, obesity, diabetes, chronic obstructive pulmonary disease, asthma, and reproductive health issues.^45^, ^46^, ^47^, ^56^, ^59^ Lastly, four studies examined survivorship burdens related to treatment specific toxicities with anticancer therapies captured with clinical assessments and chart review. Examples include methotrexate and mucositis, anthracyclines and cardiomyopathy, ototoxicity and cisplatin, and chemotherapy with gastrointestinal distress (Figure 3).^42^, ^44^, ^50^, ^53^, ^54^, ^55^, ^60^

### 4.4 DNA methylation measurements

Of the 22 studies evaluated, eight (31.8%) were longitudinal in nature with multiple DNAm measurements at multiple time points^40^, ^42^, ^43^, ^51^, ^57^, ^58^, ^60^ (Figure 2; Table 2; Table S3). Relative to time of anti‐cancer therapy initiation, 11 studies measured DNAm within 3 months,^40^, ^42^, ^43^, ^50^, ^51^, ^52^, ^53^, ^54^, ^57^, ^58^, ^60^ while seven studies had measurement between 3 months to 1 year^40^, ^42^, ^43^, ^44^, ^51^, ^57^, ^58^ (Table 2; Table S3). Most studies have DNAm measurements over 1 year removed from initiation of anti‐cancer therapy (Table 2; Table S3). Relative to therapy course, eight studies collect samples prior to therapy in longitudinal study designs (Table 2; Table S3).^40^, ^41^, ^42^, ^43^, ^51^, ^57^, ^58^, ^60^ Eight studies also collect samples during active anti‐cancer treatment,^42^, ^44^, ^50^, ^51^, ^52^, ^53^, ^57^, ^58^ with 3 collecting them immediately after completion of treatment.^40^, ^51^, ^54^, ^60^ Most studies collect samples more than 1 month removed therapy completion. With respect to DNA methylation measurement platform, 21 of 22 studies utilized Ilumina Epic or 450 K arrays to perform methylation measurements (Table S3).

**TABLE 2:** DNA methylation data utilized in studies (n=22).

Category	Count	Percentages
Number of methylation measurements		
Cross‐sectional study	14	63.6
Longitudinal study	8	36.4
Measurement timing: therapy initiation		
Within 3 months of therapy	11	50.0
Within 1 year of therapy	7	31.8
More than 1 year removed from therapy	14	63.6
Measurement timing: Therapy course		
Prior to treatment	8	31.8
During treatment	8	31.8
Immediately following treatment	3	13.6
1+ months removed from treatment	18	81.8
Methylation data used		
Epigenetic clocks	10	45.5
Differentially methylated CpG sites	12	54.5
Epigenetic aging studies (*n* = 10)		
Horvath	7	70.0
Extrinsic epigenetic age	4	40.0
PhenoAge	5	50.0
Hannum	4	40.0
GrimAge	5	50.0
DunedinPace	3	30.0
Multiple epigenetic clocks	8	80.0
Residuals to chronological age	7	70.0
Differential DNA methylation studies (*n* = 12)		
Epigenome wide association studies	5	41.7
CpGs within genes of interest	4	33.3
CpGs differentially methylated over time	2	16.7
CpGs differentially methylated based on therapy	1	8.3

*Note*: An overview of methylation measurement approaches utilized within included studies. This includes the number of methylation measurements performed for each patient, the timing of methylation measurements, and the type of methylation data utilized. Information specific to Epigenetic aging studies and differential DNA methylation is also captured. For all categories, the count column shows the numerical count of studies matching that category. Percentages reported are out of the total number of studies (*n* = 22) except for categories specific to epigenetic aging studies and differential DNA methylation studies where percentages are based on the number of studies within those sub‐categories.

Epigenetic clocks were utilized in 10 (45.5%) of the studies collected.^41^, ^42^, ^44^, ^45^, ^49^, ^51^, ^57^, ^58^, ^59^, ^60^ Within these studies, most of them utilized multiple epigenetic clocks for comparison (Table 2). The most utilized epigenetic clock throughout these studies was Horvath's in seven (70%) different studies, followed by PhenoAge and GrimAge utilized five (50%) times each, with Hannum and DunedinPace metrics each being used four (40%) and three (30%) times respectively. In addition to considering measured epigenetic age, seven (70%) studies also considered the residual value of epigenetic age to chronological age (Table 2).

Differentially Methylated CpG sites were considered in 12 (54.5%) of the collected studies.^19^, ^40^, ^43^, ^46^, ^47^, ^48^, ^50^, ^52^, ^53^, ^54^, ^55^, ^56^ Epigenome wide association studies were performed in five (41.7%) of these studies,^46^, ^48^, ^54^, ^55^, ^56^ while four studies (33.3%) limited CpG sites to genomic sequences of interest identified prior.^19^, ^50^, ^52^, ^53^ Examples of this include utilization of CpG sites associated with within molecular pathways of interest, CpG sites only associated promoter sequences, or even only considering CpG sites within genes having significantly altered expression as demonstrated by prior experiments or research. Two studies (16.7%) limited considered CpG sites to those that are differentially expressed over time.^40^, ^43^ Finally, one (8.3%) study limited considered CpG sites to those that differentially expressed based on various treatment exposures.^47^ Within studies considering methylation levels at individual sites, four (18.2%) also performed analysis to discover differentially methylated regions of CpG site clusters.^48^, ^50^, ^55^, ^56^

### 4.5 Multivariate modeling approaches with DNA methylation data

With respect to multivariate models utilizing epigenetic age data, various approaches were described including both linear and logistic regressions, exponential regression, mixed effects regression, and generalized estimating equations. Common covariates considered within these models included chronological age, chemotherapy agent exposure, race/ethnicity, radiation therapy exposure, and BMI (Figure 2A). Other covariates considered in select models included gender, general comorbidities or a composite comorbidity score, blood cell composition, smoking status, cancer staging, HPV status, education level, and marital status (Figure 2A). In most studies, epigenetic age metrics were considered as covariates with other meaningful data predicting survivorship burden outcomes as a response variable. There was a singular study due to limited sample size that did not consider a multivariate approach, but instead utilized Spearman's rank correlation coefficients to assess associations between epigenetic age and measures of interest.

Multivariate models utilized with individual CpG site beta values included multiple linear regression and empirical Bayes models commonly implemented in limma software tools.^61^ Covariates considered in multivariate models with CpG methylation levels included age, blood cell composition, chemotherapy and radiation therapy exposures, body mass index, general comorbidities or a composite comorbidity score, and gender (Figure 2B). Other covariates considered include smoking status, gene expression data, race, and steroid exposure (Figure 2B). In some cases, studies considered surrogate variable techniques instead of common clinical covariates. CpG site beta vales were considered the response variable in most of these models, with the survivorship burden of interest a covariate alongside other selected variables.

Many of these studies describe first considering many covariates or predictors related to patient demographic, clinical, and laboratory values prior to implementing finalized models. Feature selection for final models assessing the significance of DNAm data included some of the following techniques: Akaike Information Criterion optimization, stepwise feature reduction, and preliminary univariate variable screening to identify significant features for multivariate models. In addition to feature selection strategies, various corrective measures to normalize measured methylation values across different samples were taken. These include batch correction techniques, surrogate variable placement, and utilizing identified principal components with gene expression data.

### 4.6 Significant associations of DNA methylation with survivorship burdens

Epigenetic age measurements had various associations with outcomes related to survivorship burdens. Two studies found significant associations between epigenetic age assessed within 3 months of anti‐cancer therapy initiation and outcomes related to survivorship burdens, those outcomes related to psychomotor speed, fatigue, grip strength, a 6‐min walk test (Table 3).^42^, ^44^, ^50^ Three studies found significant associations with epigenetic age assessed 3 months after anti‐cancer therapy initiation but prior to 1 year (Table 3). The survivorship burden outcomes with significant associations within those studies included memory, anxiety, depression, fatigue, sleep, pain, and overall quality of life (Table 3).^42^, ^51^, ^58^ Lastly, five studies found significant associations with epigenetic age assessed more than 1 year removed from anti‐cancer therapy initiation and outcomes related to survivorship burdens.^41^, ^42^, ^45^, ^49^, ^59^ Those outcomes were: cognition, memory, overall health quality, physical impairment status, vitality, social function, pain, emotional health, hypertension, myocardial infarction, obesity, chronic obstructive pulmonary disease, neuropathy, pulmonary diffusion, and cumulative chronic disease burden (Table S4).

**TABLE 3:** Significant Associations of Epigenetic Clocks with Survivorship Burdens.

	0–3 months	3 months–1 year	1+ year
Neurocognition	*Psychomotor speed* (GrimAge,^42^)	*Memory* (Horvath, IEAA,^42^) *Neurocognitive index* (EEAA, GrimAge,^42^) *Psychomotor speed* (GrimAge,^42^)	*Self‐reported cognition* (EEAA,^41^) *Memory* (Horvath,^42^)
Psychiatry		*Anxiety* (GrimAge, Horvath, IEAA,^42^) *Depression* (IEAA, GrimAge,^42^)	
General wellness	*Fatigue* (GrimAge,^42^) *Grip strength* (DunedinPace, GrimAge, PhenoAge,^44^) *6*‐*min walk test* (PhenoAge, GrimAge, DunedinPace,^44^)	*Fatigue* (GrimAge,^42^) *Sleep disturbance* (GrimAge, IEAA,^42^) *Pain* (GrimAge,^42^) *Fatigue* (Levine, Hannum,^51^) *Overall quality of life* (Levine's,^58^)	*Overall physical function* (GrimAge,^49^) *Perceived Impairment* (GrimAge, AgeAccelPheno,^49^) *Global health* (AgeAccelPheno, DunedinPace,^49^) *Vitality* (AgeAccelPheno,^49^) *Social function* (AgeAccelPheno,^49^) *Pain* (AgeAccelPheno,^49^) *Emotional health* (AgeAccelPheno,^49^)
Chronic conditions			*Hypertension* (EAA,^45^) *Myocardial infarction* (EAA,^45^) *Obesity* (EAA,^45^) *COPD* (EAA,^45^) *Peripheral motor neuropathy* (EAA,^45^) *Peripheral sensory neuropathy* (EAA,^45^) *Pulmonary diffusion* (EAA,^45^) *Obesity* (Levine's, GrimAge, Horvath,^59^) *Cumulative disease burden* (Levine's, GrimAge, Hannum,^59^)

*Note*: Each column represents the time frame DNA methylation was measured relative to cancer therapy initiation with time frames of within 3 months, between 3 months and 1 year, and over 1 year removed. Each row represents a survivorship burden domain. Entries represent significant associations of epigenetic age with survivorship burdens in the following format: Survivorship burden outcome (epigenetic clock(s), citation number).

Abbreviations: EAA, epigenetic age acceleration; EEAA, extrinsic epigenetic age acceleration; IEAA, intrinsic epigenetic age acceleration.

Various associations were also found with respect to methylation levels at individual CpG sites and outcomes related to survivorship burdens. Four studies measuring DNAm within 3 months of anticancer therapy initiation found significant differentially methylated CpGs and differentially methylated regions associated with survivorship burden outcomes (Table 4).^40^, ^50^, ^52^, ^53^ Those outcomes were self‐perceived cognitive impairment, psychological symptoms, fatigue, and gastrointestinal symptoms. Two studies measuring DNAm 3 months after anticancer therapy initiation but prior to 1 year removed found significant differentially methylated CpGs associated with survivorship burden outcomes (Table 4).^19^, ^43^ Those outcomes were memory and peripheral neuropathy. Lastly, four studies assessing DNAm more than 1 year removed from anticancer therapy initiation found significant differentially methylated CpGs and significant differentially methylated regions associated with survivorship burden outcomes (Table 4).^46^, ^48^, ^54^, ^55^ Those outcomes were educational attainment, personal income, area deprivation index, lipid measurements, ototoxicity, and cardiomyopathy (Table S4).

**TABLE 4:** Significant associations of differentially methylated CpG sites and regions with survivorship burdens.

	0–3 months	3 months–1 year	1+ Year
Neurocognition	1, self‐perceived cognition^(^ ^40^ ^)^	56, memory^(^ ^43^ ^)^	
Psychiatric	6, psychological symptom cluster^(^ ^52^ ^)^		
General wellness	23, fatigue^(^ ^50^ ^)^	8, peripheral neuropathy^(^ ^19^ ^)^	88, educational attainment^(^ ^48^ ^)^ 23, personal income^(^ ^48^ ^)^ 19, area deprivation index^(^ ^48^ ^)^
Chronic conditions			2, high‐density lipoprotein^(^ ^46^ ^)^ 2, total cholesterol^(^ ^46^ ^)^ 31, triglycerides^(^ ^46^ ^)^ 1, low‐density lipoprotein^(^ ^46^ ^)^ 63, hyper‐cholesteralemia^(^ ^47^ ^)^ 7, obesity^(^ ^47^ ^)^ 16, hypertriglyceridemia^(^ ^47^ ^)^
Treatment specific Toxicities	1, GI symptom cluster^(^ ^15^ ^)^		6, ototoxicity^(^ ^54^ ^)^ 2, cardiomyopathy^(^ ^55^ ^)^

*Note*: Each column represents the time frame DNA methylation was measured relative to cancer therapy initiation with time frames of within 3 months, between 3 months and 1 year, and over 1 year removed. Each row represents a survivorship burden domain. Entries represent significant differentially methylated individual CpG sites and regions with survivorship burdens in the following format: Number of differentially methylated sites, survivorship burden outcome (citation number).

## 5 DISCUSSION

In this scoping review, we observed a growing body of literature examining the association of cancer survivorship burdens and DNAm measurements from peripheral blood samples. Most of the discovered studies were published within the past 5 years, demonstrating recent growing interest in utilizing DNAm data to gain insight into survivorship issues. It is important to note that there was significant variety with respect to the cancer survivor populations considered, survivorship burden outcomes assessed, DNAm measurement strategies utilized, and multivariate modeling approaches implemented. Many significant associations between survivorship burdens and measured peripheral blood DNAm of cancer survivors were identified. The findings of this review highlight the potential of DNAm measurements and how they can better inform researchers on the underlying causes of survivorship burdens or identify targets for intervention. However, significant differences amongst the collected studies limit not only direct comparison of outcomes, but also assessments of overall clinical utility of DNAm data. Their heterogeneous nature also makes it challenging to reach consensus with respect to best practices of utilizing DNAm measurements in survivorship research and clinical care.

There are noticeable trends with respect to covariates considered when evaluating the extent DNAm data are predictive of survivorship burdens. Efforts were made to incorporate clinically relevant information known to influence outcomes of interest. For instance, current diagnoses of anxiety and/or depression were often considered with outcomes related to neurocognition or fatigue. Studies with quality‐of‐life metrics or assessing social determinants of health often included BMI due to its known influence. Covariates were also considered having known influences on DNA methylation measurements. These include many patient demographic factors such as age, ethnic background, smoking history and gender.^62^, ^63^, ^64^, ^65^, ^66^ Additional anti‐cancer treatment factors were selected known to influence DNAm, including specific therapeutic agents used and cumulative therapy exposure. Cellular composition of samples was also considered; as diverse cell types can have distinct epigenetic signatures.^67^, ^68^, ^69^ Future studies should consider whether to include these confounders within their analyses.

Most studies utilizing epigenetic clocks implemented multiple of them, and they utilized residual values of epigenetic age relative to chronological age to account for the confounding effect of chronological aging with these metrics.^70^, ^71^ Significant associations between epigenetic aging and a variety of survivorship burdens were discovered at a broad range of timepoints relative to anti‐cancer therapy initiation. For instance, multiple studies identified significant associations of accelerated epigenetic aging measured within 3 months of therapy initiation and various aspects of physical health (fatigue, physical strength measures).^42^, ^44^ Further removed from therapy initiation, significant associations of epigenetic age were identified with different psychiatric symptoms (anxiety, depression).^58^ Even multiple years removed from chemotherapy course, the occurrence of several chronic health conditions and quality of life metrics had significant ties to accelerated epigenetic aging.^45^, ^59^ Some studies suggested some epigenetic aging metrics may be more appropriate for these analyses. Epigenetic clocks like GrimAge and PhenoAge were designed emphasizing CpG sites representative of biomarkers, comorbidities, and lifestyle factors capturing mortality risk.^72^ While the strength of mortality risk predictions with GrimAge are well established, potential associations with onset of different survivorship issues would make sense given their design and likely association of these symptoms with mortality risk. Other clocks like Horvath's were developed more strictly to predict chronological age in diverse samples regardless of overall health status.^58^, ^73^ However, while epigenetic aging continues to be explored in this space it is important to assess each metric of aging to see which ones are most informative with respect to survivorship issues.

Evaluation of the location(s) of differentially methylated CpG sites and regions provided insights into underlying biologic causes of survivorship issues. For instance, epigenome wide association studies evaluating impaired cognition showed significant positions in genes related to inflammation.^40^, ^43^ Studies evaluating long term associations of cardiovascular outcomes highlighted differentially methylated sites in genes associated with lipid metabolism and production.^47^ Significant positions were identified comparing populations of cancer survivors experiencing different levels of educational attainment and income status, which highlighted genes having established associations with cognition and different environmental exposures such as smoking.^48^ Key CpG sites identified in some studies were utilized in pathway analyses to better interpret methylation data from several different sites at once, which highlighted immune and inflammatory pathways in various etiologies.^53^, ^55^, ^56^ The exact mechanisms by which DNA methylation would influence survivorship outcomes is not perfectly understood; however multiple authors suggested its influence on gene expression plays a meaningful role.

Different approaches were implemented after identifying significant CpG sites to further validate findings. Downstream biomarkers were used as validation in some cases, utilizing gene expression data to assess the extent methylation levels influenced measured expression for corresponding genes.^19^, ^46^, ^48^ Validation of significant CpGs also occurred by measuring methylation within multiple platforms or observing if methylation changes are matched in salivary samples.^52^, ^54^ To demonstrate practical utility and the strength of DNAm associations with survivorship burdens, future studies will need reserved cohorts to validate observed findings which wasn't explicitly observed in any study designs. The development of the epigenetics clocks previously described are an example of effective experimental design, which leaned on publicly available databases to validate their robust predictions. Publicly available databases have allowed tremendous progress with various elements of cancer and tumor genomics; further development of these systems to capture more detailed clinical outcomes can allow for similar progress in cancer survivorship research.

While this review focuses human studies, much can be learned from pre‐clinical models as DNAm is explored as a biomarker in survivorship care. Researchers can measure diverse chemotherapy‐induced symptoms in mice that reflect many chemotherapy toxicities and survivorship burdens such as gastrointestinal distress, pain, cognitive function, and fatigue.^74^, ^75^, ^76^, ^77^ Genes having significantly altered methylation status relative to symptomatic burden can be identified in chemotherapy treated mice, leading to new hypotheses and better justifying larger scale studies in human patients. Additionally, preliminary discoveries in human studies can be validated in animal models as additional evidence for suspected DNAm involvement in survivorship symptoms. Given challenges and costs of creating cohorts of cancer survivors with needed symptomatic data, and the relative infancy of this research, animal models maybe a practical approach create more rapid progress in this research.

There is a growing body of evidence that supports associations of DNAm patterns and health outcomes related to survivorship. Associations of epigenetics and cognitive function have been identified in the elderly and in disease states such as dementia or Alzheimer's.^78^, ^79^, ^80^ More examples include identified DNAm associations with of depression, fatigue, pain, and cardiovascular comorbidities.^81^, ^82^, ^83^, ^84^, ^85^ Given the known associations of DNAm with various clinical outcomes already, steps with respect to patient sample selection can be taken in DNAm studies to ensure observed differences are specific to cancer survivorship. Population matching approaches can incorporate factors known to influence DNAm and observed clinical outcomes such as patient demographics, comorbidities, therapy exposures, and cancer progression. Given the challenging nature of creating robust patient populations having DNAm data, utilization of appropriate matched controls for the intended research question can facilitate more meaningful findings.

Another area of consideration with respect to study designs is the timing and frequency of DNAm measurements. A number of longitudinal studies in this review observed changes in DNAm at individual genomic sites and increases in epigenetic age just a couple of months removed from therapy initiation.^40^, ^58^ Additionally, multiple studies also showed persistent changes over multiple years, or significant differences between cancer survivors and matched healthy controls many years removed from their active therapy.^45^, ^55^ Many factors can influence the methylome of cancer survivors over time; one example being patients placed on exercise regimens having different DNAm patterns and epigenetic aging compared those that do not.^86^, ^87^, ^88^ What might be more practical in selecting measurement timing, given unknown potential variability of DNAm over time, is to select measurement time points specific to when clinical outcomes are expected to occur. Having longitudinal studies with DNAm measurements prior to therapy initiation and follow up measurements timed with relevant clinical assessments is an ideal approach. This presents a unique challenge with clinical outcomes having variable or unknown time to onset, which would necessitate multiple clinical assessments and DNAm measurements.

We have generated a list of recommendations that investigators should adopt when they design a study from the ground up to evaluate epigenetic changes and survivorship health burden (Table 5). With respect to initial study populations recruited and study design, investigators can emphasize recruiting appropriately matched controls based on study objectives. Investigators can also emphasize patient populations with similar anti‐cancer therapy exposures to better attribute methylation changes to these agents, especially newer less investigated targeted agents like immune checkpoint inhibitors and tyrosine kinase inhibitors. Longitudinal DNAm measurements before and after anti‐cancer therapy exposures will allow insight into survivorship specific DNAm changes. Analytical methods should account for known confounders influencing DNAm and compare their predictive potential of survivorship issues relative to epigenetic markers discovered. To give context to significant findings, follow up bioinformatics analysis can demonstrate how significant changes in DNAm and epigenetic aging manifest altering downstream biomarkers. Eventually, studies can further validate the associations of DNAm with clinically meaningful outcomes by utilizing reserved testing data sets from separate patient populations to support their findings.

**TABLE 5:** Recommendations for future studies evaluating epigenetics and survivorship issues in peripheral blood samples.

Recommendations
Include a matched control arm appropriate for specific study goals to limit confounding of observed DNA methylation differences Consider patient populations with similar anti‐cancer therapy exposures, especially less investigated targeted agents like immune checkpoint inhibitors or tyrosine kinase inhibitors, to better attribute methylation changes to specific therapeutic agents. Perform longitudinal DNA methylation measurements including measurements prior to anti‐cancer therapy exposure and follow up measurements consistent with expected timing of survivorship burdens of interest Account for confounding factors that influence epigenetic patterns including age, sex, smoking history, ethnicity, and comorbidities Implement several different epigenetic clocks until the best metric(s) capturing epigenetic age acceleration in cancer patients are more established Provide context for significant associations of DNAm and survivorship with follow up downstream bio‐informatics studies (RNA and Protein expression) Validate observed associations of DNAm and survivorship burdens in reserved testing populations or follow‐up prospective studies

There are limitations to this review. Our literature search procedure only utilized a single search engine, and multiple studies were discovered with manual search methods. As interest in the utility of DNA methylation patterns in survivorship care grows, this study can justify larger systematic reviews. Single cohorts of patients from certain trials are represented in multiple publications, with each publication evaluating different outcomes or utilizing different analytical methods. The criteria for each prospective study to identify significant associations with DNAm and survivorship burdens varied. Analyses within these studies encompassed different multivariate modeling approaches, different covariates, and had different population subtypes and sizes. Many of the studies had distinct experimental and analytical steps before and after assessing associations of DNAm data with survivorship burdens. Those are not represented in this review and may provide additional context to their findings. All these factors make it challenging to compare identified associations across studies. With respect to DNAm data, studies within this review all used measurements from chips or arrays.

We observe that there is evidence of associations of DNAm patterns in peripheral blood samples in cancer survivors being associated with a variety of survivorship burdens. Given the current body of evidence, DNAm may serve as a useful biomarker in the future to identify cancer patients at risk of many survivorship issues. Additional studies informing researchers on the underlying causes of survivorship issues may lead to better management strategies in the future.

## AUTHOR CONTRIBUTIONS

**Michael Sayer:** Conceptualization (equal); data curation (equal); formal analysis (equal); methodology (equal); visualization (equal); writing – original draft (equal); writing – review and editing (equal). **Ding Quan Ng:** Conceptualization (equal); data curation (equal); formal analysis (equal); methodology (equal); visualization (equal); writing – original draft (equal); writing – review and editing (equal). **Raymond Chan:** Conceptualization (equal); methodology (equal); project administration (equal); validation (equal); writing – review and editing (equal). **Kord Kober:** Conceptualization (equal); methodology (equal); project administration (equal); validation (equal); writing – review and editing (equal). **Alexandre Chan:** Conceptualization (equal); methodology (equal); project administration (equal); supervision (equal); validation (equal); writing – original draft (equal); writing – review and editing (equal).

## CONFLICT OF INTEREST STATEMENT

The authors declare there is no conflict regarding the publication of this study with respect to their financial or institutional interests.

## PRÉCIS

Our literature review collected studies examining associations between various forms of DNA methylation data and survivorship issues experienced by cancer survivors. Significant associations with both epigenetic aging metrics and differentially methylated DNA sites with survivorship issues were discovered in various studies, with each one having distinct patient populations, study designs, and analytical approaches.

## Supporting information


Table S1.

## Data Availability

Data sharing is not applicable to this article as no new data were created or analyzed in this study.
