# Supplementary material for: Current evidence supporting associations of DNA methylation measurements with survivorship burdens in cancer survivors: A scoping review
Source: Cancer Med. 2024 Jul 4;13(13):e7470. doi: 10.1002/cam4.7470 (PMC11222976; doi:10.1002/cam4.7470)
Supplement: Supplementary file 1 — Table S1. [file CAM4-13-e7470-s001.docx]

**Supplemental Tables:**

**Supplemental Table 1: Keyword Searches Utilized for PubMed Search**

Each row represents a combination of keywords utilized together in PubMed searches to identify potential studies. The first column represents the search number in the order they were performed, followed by keywords utilized for each prospective search in keyword columns 1, 2, and 3.

| **Search Number** | **Keyword 1** | **Keyword 2** | **Keyword 3** |
| --- | --- | --- | --- |
| 1 | Cancer | Differential DNA Methylation | Quality of Life |
| 2 | Cancer | Differential DNA Methylation | Side Effects |
| 3 | Cancer | Differential DNA Methylation | Survivorship |
| 4 | Cancer | DNA Methylation | Quality of Life |
| 5 | Cancer | DNA Methylation | Side Effects |
| 6 | Cancer | DNA Methylation | Survivorship |
| 7 | Cancer | Epigenetic Aging | Quality of Life |
| 8 | Cancer | Epigenetic Aging | Side Effects |
| 9 | Cancer | Epigenetic Aging | Survivorship |
| 10 | Cancer Survivor | Differential DNA Methylation |  |
| 11 | Cancer Survivor | DNA Methylation |  |
| 12 | Cancer Survivor | Epigenetic aging |  |
| 13 | Cancer Treatment | Differential DNA Methylation | survivorship |
| 14 | Cancer Treatment | Differential DNA methylation | quality of life |
| 15 | Cancer Treatment | Differential DNA methylation | side effects |
| 16 | Cancer Treatment | DNA methylation | quality of life |
| 17 | Cancer Treatment | DNA methylation | side effects |
| 18 | Cancer Treatment | DNA Methylation | survivorship |
| 19 | Cancer Treatment | Epigenetic aging | quality of life |
| 20 | Cancer Treatment | Epigenetic aging | side effects |
| 21 | Cancer Treatment | Epigenetic aging | survivorship |
| 22 | Chemotherapy | Differential DNA Methylation | quality of life |
| 23 | Chemotherapy | Differential DNA Methylation | Side effects |
| 24 | Chemotherapy | Differential DNA Methylation | survivorship |
| 25 | Chemotherapy | DNA Methylation | quality of life |
| 26 | Chemotherapy | DNA Methylation | Side effects |
| 27 | Chemotherapy | DNA Methylation | survivorship |
| 28 | Chemotherapy | Epigenetic aging | quality of life |
| 29 | Chemotherapy | Epigenetic aging | Side Effects |
| 30 | Chemotherapy | Epigenetic aging | survivorship |

**Supplemental Table 2: Summary of Epigenetic Clocks**

From left to right, the columns represent the short name for the epigenetic clock, followed by its predicted outcome, the number of CpG sites it utilizes, the criteria for considered CpG sites for each metric, and a brief description of the sample used.

| **Epigenetic Clock** | **Predicted**  **Outcome** | **CpG sites** | **CpG Selection Criteria** | **Patient Sample description** |
| --- | --- | --- | --- | --- |
| *Horvath* | Age | 353 | Non-specific CpG sites | 7,844 samples of various somatic tissues from TCGA data sets |
| *Intrinsic Epigenetic Age*  *(Derived from Horvath)* | Age | 353 | Non-specific CpG sites | Same as above, uses white blood cell counts to limit confounding from changing cell composition with ageing |
| *Hannum* | Age | 71 | Non-specific CpG sites | 656 whole blood patient samples from trial participants. |
| *Extrinsic Epigenetic Age*  *(Derived form Hannum)* | Age | 71 | Non-specific CpG sites | Same as above, weights white blood cell counts to represent immune specific ageing |
| *Levine or PhenoAge* | Mortality Risk and Age | 513 | CpG sites were selected best predicting assigned mortality risk derived from measured clinical biomarkers representative of common comorbidities | 9,936 whole blood samples from 5 different studies |
| *GrimAge* | Mortality Risk and Age | 1030 | CpG sites directly associated with smoking pack years and plasma protein levels having known associations with mortality risk. | 1,731 whole blood samples from the Farmington Heart Study |
| *DunedinPace* | Rate of Biological Ageing | 173 | CpG sites associated with biomarkers describing overall health of cardiovascular, metabolic, renal, hepatic, immune, dental, and pulmonary organ systems | 1037 whole blood samples from the Dunedin study |

**Supplemental Table 3: Citations of Studies Utilized for Review**

Each column from left to right includes the citation number referencing each study, the study title, lead author last name and publication year, the type of DNA methylation data utilized in the study, the DNA methylation measurement platform, and a brief description of the study design with respect to capturing blood samples used for DNA methylation measurements.

| **#** | **Study Title** | **Author & Year** | **Cancer Patient Population** | **Methylation Data** | **Measurement Platform** | **Sampling Description** |
| --- | --- | --- | --- | --- | --- | --- |
| **19** | Differential methylation and expression of genes in the hypoxia-inducible factor 1 signaling pathway are associated with paclitaxel-induced peripheral neuropathy in breast cancer survivors and with preclinical models of chemotherapy-induced neuropathic pain. | Kober,  2020 | 50 Breast cancer survivors | Differential Methylation of CpG sites/Regions | Illumina Methylation EPIC BeadChip array | Blood samples were of were obtained from patients at least 3 months removed from completion of platinum and/or taxane based chemotherapy regimen |
| **40** | Impact of chemotherapy for breast cancer on leukocyte DNA methylation landscape and cognitive function: a prospective study. | Yao,  2019 | 93 Non-metastatic breast cancer patients | Differential Methylation of CpG sites/Regions | Illumina Methylation EPIC BeadChip array | Blood samples for DNA methylation assessments were taken before and immediately after completion of chemotherapy regimen. |
| **41** | Epigenetic aging in older breast cancer survivors and non-cancer controls: preliminary findings from the Thinking and Living with Cancer (TLC) Study. | Rentscher,  2023 | 93 Non-metastatic breast cancer patients | Epigenetic Ageing | Illumina Methylation EPIC BeadChip array | Blood samples were taken prior to systemic therapy and 24 to 60 months removed from initiation of systemic therapy |
| **42** | Exploring the relationship between DNA methylation age measures and psychoneurological symptoms in women with early-stage breast cancer. | Yang,  2022 | 72 Early stage breast cancer patients | Epigenetic Ageing | Infinium Human Methylation 450K BeadChip array | Blood samples were taken at 5 timepoints. T1- before the receipt of chemotherapy ~ 1 month removed from initiation, T2- Midpoint of chemotherapy, T3- 6 months after chemotherapy initiatoin, T4- 12 months after chemotherapy initiation, and T5- 24 months after the initiation of chemotherapy |
| **43** | Differential DNA methylation following chemotherapy for breast cancer is associated with lack of memory improvement at one year. | Yang,  2020 | 75 Early stage breast cancer patients | Differential Methylation of CpG sites/Regions | Infinium Human Methylation 450K BeadChip array | Blood samples were utilized from 2 timepoints, one before the receipt of chemotherapy and one 12 months after chemotherapy initiation |
| **44** | Exercise and epigenetic ages in older adults with myeloid malignancies. | Loh,  2023 | 20 Cancer patients with myeloid malignancies | Epigenetic Ageing | Illumina Methylation EPIC BeadChip array | Two Blood samples were collected 8 weeks apart during active chemotherapy, both during active outpatient chemotherapy |
| **45** | Epigenetic Age Acceleration and Chronic Health Conditions Among Adult Survivors of Childhood Cancer. | Qin,  2021 | 1563 Childhood cancer survivors | Epigenetic Ageing | Illumina Methylation EPIC BeadChip array | Blood samples for children’s cancer survivors were taken at least 10 years removed from initial cancer diagnosis and when subjects were 18 years of age and older. |
| **46** | Distinct DNA methylation signatures associated with blood lipids as exposures or outcomes among survivors of childhood cancer: a report from the St. Jude lifetime cohort. | Dong,  2023 | 2,422 Childhood cancer survivors | Differential Methylation of CpG sites/Regions | Illumina Methylation EPIC BeadChip array | Blood samples for children’s cancer survivors were taken at least 10 years removed from initial cancer diagnosis and when subjects were 18 years of age and older. |
| **47** | Persistent variations of blood DNA methylation associated with treatment exposures and risk for cardiometabolic outcomes in long-term survivors of childhood cancer in the St. Jude Lifetime Cohort. | Song,  2021 | 2,052 Childhood cancer survivors | Differential Methylation of CpG sites/Regions | Illumina Methylation EPIC BeadChip array | Blood samples for children’s cancer survivors were taken at least 10 years removed from initial cancer diagnosis and when subjects were 18 years of age and older. |
| **48** | Blood DNA methylation signatures are associated with social determinants of health among survivors of childhood cancer. | Song,  2022 | 1,876 Childhood cancer survivors | Differential Methylation of CpG sites/Regions | Illumina Methylation EPIC BeadChip array | Blood samples for children’s cancer survivors were taken at least 10 years removed from initial cancer diagnosis and when subjects were 18 years of age and older. |
| **49** | Accelerated epigenetic aging and myopenia in young adult cancer survivors. | Gehle,  2023 | 58 Young adult cancer survivors | Epigenetic Ageing | Illumina Methylation EPIC BeadChip array | Blood samples were retrieved many years removed from completion of therapy |
| **50** | A Pilot Study Using a Multistaged Integrated Analysis of Gene Expression and Methylation to Evaluate Mechanisms for Evening Fatigue in Women Who Received Chemotherapy for Breast Cancer. | Flowers,  2019 | 36 Breast cancer patients | Differential Methylation of CpG sites/Regions | Infinium Human Methylation 450K BeadChip array | Blood samples were obtained from patients whom had received chemotherapy within 4 weeks prior and were scheduled to receive at least 2 more cycles. |
| **51** | Epigenetic age acceleration, fatigue, and inflammation in patients undergoing radiation therapy for head and neck cancer: A longitudinal study. | Xiao,  2021 | 133 Non-metastatic head and neck cancer patients | Epigenetic Ageing | Illumina Methylation EPIC BeadChip | Blood samples were obtained from patients at the following timepoints: before radiotherapy, immediately completing radiotherapy, 6 months post radiotherapy, and 12 months post radiotherapy. |
| **52** | Epigenetic Regulation of Inflammatory Mechanisms and a Psychological Symptom Cluster in Patients Receiving Chemotherapy. | Harris,  2023 | 1,071 Cancer patients | Differential Methylation of CpG sites/Regions | Illumina Infinium 450K and EPIC array | Blood samples were provided by patients 1 month removed from their most recent chemotherapy cycle with at least 2 more cycles scheduled. |
| **53** | Gastrointestinal Symptom Cluster is Associated With Epigenetic Regulation of Lymphotoxin Beta in Oncology Patients Receiving Chemotherapy. | Harris,  2023 | 1,071 Cancer patients | Differential Methylation of CpG sites/Regions | Illumina Infinium 450K and EPIC microarrays | Blood samples were provided by patients 1 month removed from their most recent chemotherapy cycle with at least 2 more cycles scheduled. |
| **54** | DNA methylation of a novel PAK4 locus influences ototoxicity susceptibility following cisplatin and radiation therapy for pediatric embryonal tumors. | Brown,  2017 | 80 Childhood cancer patients | Differential Methylation of CpG sites/Regions | Infinium Human Methylation 450K BeadChip array | Blood and salivary samples were collected immediately following completion of cisplatin regimens |
| **55** | Identification of novel hypermethylated or hypomethylated CpG sites and genes associated with anthracycline-induced cardiomyopathy. | Singh,  2023 | 104 Cancer survivors | Differential Methylation of CpG sites/Regions | Illumina Methylation EPIC BeadChip array | Blood samples were collected years removed from completion of anthracycline therapy |
| **56** | Cisplatin treatment of testicular cancer patients introduces long-term changes in the epigenome. | Bucher-Johannessen,  2019 | 279 Testicular cancer survivors | Differential Methylation of CpG sites/Regions | Illumina Methylation EPIC BeadChip array | Blood samples were collected years removed from completion of treatment |
| **57** | Longitudinal Analysis of Sleep Disturbance in Breast Cancer Survivors. | Yang,  2023 | 74 Early stage breast cancer patients | Epigenetic Ageing | Infinium Human Methylation 450K BeadChip array | Blood samples were taken at 5 timepoints. T1- before the receipt of chemotherapy ~ 1 month removed from initiation, T2- Midpoint of chemotherapy, T3- 6 months after chemotherapy initiation, T4- 12 months after chemotherapy initiation, and T5- 24 months after the initiation of chemotherapy |
| **58** | Association of epigenetic age acceleration with risk factors, survival, and quality of life in patients with head and neck cancer. | Xiao,  2021 | 146 Head and neck cancer patients | Epigenetic Ageing | Illumina Methylation EPIC BeadChip array | Blood samples were obtained from patients at the following timepoints: before radiotherapy, immediately completing radiotherapy, 6 months post radiotherapy, and 12 months post radiotherapy. |
| **59** | Epigenetic Age in Peripheral Blood Among Children, Adolescent, and Adult Survivors of Childhood Cancer. | Plonski,  2023 | 2846 Childhood cancer survivors | Epigenetic Ageing | Illumina Methylation EPIC BeadChip array | Blood samples for children’s cancer survivors were taken at least 10 years removed from initial cancer diagnosis and when subjects were 18 years of age and older. |
| **60** | Global methylation in relation to methotrexate-induced oral mucositis in children with acute lymphoblastic leukemia | Oosterom,  2018 | 82 Pediatric acute lymphoblastic leukemia patients | Global DNA Methylation/Epigenetic Ageing | Zymo Easy DNA methylation Kit | Blood samples were collected before and after high dose methotrexate regimens |

**Supplemental Table 4: Examples of Statistics Representing Significant Findings In Evaluated Studies**

Each column from left to right includes the citation number, the sample statistic representing a significant result, the statistical methodology employed, and a brief description of it’s interpretation. These entries are intended to represent single examples of significant findings from each study for the reader, not intended to be cumulatively representative of statistics for all significant findings.

| **#** | **Statistic** | **Statistical Methodology** | **Description of Result** |
| --- | --- | --- | --- |
| 19 | Log Fold Change= −0.011,  p= 1.95e-5 | Limma regression, reported as log-fold change. | Beta values for CpG site cg27335386 for patients experiencing paclitaxel induced peripheral neuropathy were significantly different than those that did not. |
| 40 | No coefficient reported  p= 0.04 | Linear regression | Change in beta value of CpG site 16936953 was significantly associated with change in fact-cog score. |
| 41 | 95% CI for HR: (-1.28) - (-0.01)  , p= 0.047 | Linear regression,  Reported as hazard ratio | Higher extrinsic epigenetic age value was significantly associated with lower reported perceived cognitive impairment score. |
| 42 | Coefficient =  0.799,  p= 0.031 | Linear regression models  Reported as coefficients | Higher accelerated epigenetic ageing (residuals of epigenetic age to chronological age) for GrimAge values were significantly associated with increased reported Brief Fatigue Inventory (BFI) score. |
| 43 | Coefficient = (−230.5),  p= 1.19 × 10^−5^ | Linear regression models,  Reported as coefficients | Change in beta value of CpG site cg01333080 was significantly associated with change in memory score (CNS Vital Signs). |
| 44 | Correlation Coefficient= (-0.50),  p= <0.05 | Wilcoxon ranked sums test,  Reported as correlation coefficient | Decrease in accelerated epigenetic ageing (residuals of epigenetic age to chronological age) for GrimAge was significantly associated with increased performance in 6 minute walk tests. |
| 45 | Relative Risk= 1.83,  p= 0.005 | Logistic regression,  reported as relative risk | Patients in the highest tertile of observed accelerated epigenetic ageing values relative to the entire study population for Levine’s age were significantly more likely to experience a hypertension diagnosis. |
| 46 | Effect Size = 7.03,  P=1.03e-12 | Limma regression,  reported as effect size | Beta values of CpG site cg21750129 were significantly associated with measured HDL levels. |
| 47 | Coefficient = (-2.04),  p= 6.07e-5 | Linear regression,  reported as coefficient | Lower beta values of CpG site cg19634849 had significant associations with increased risk of hypertriglyceridemia |
| 48 | Coefficient= 0.018,  P= 1.9e-13 | Linear regression,  reported as coefficient | \| Beta values of CpG site cg01731783 had significant associations with level of educational attainment \|  \| \| --- \| --- \| |
| 49 | Accelerated group average score= 77,  Non-accelerated group average score= 90,  p= <0.05 | Students T-test,  reported as a comparison of averages between two groups | Average physical function subscale score was on average significantly lower in patients experiencing accelerated epigenetic ageing (based on residuals to chronological age) based on Levine’s age. |
| 50 | Coefficient = 0.02  p= <0.04 | Limma regression,  reported as coefficient | Beta values in CpG site cg16326819 were significantly higher in patients experiencing severe fatigue compared to those experiencing moderate or mild fatigue. |
| 51 | Coefficient = 0.443,  P=0.003 | Generalized estimating linear models,  reported as an estimate (coefficient) and p-value | Higher accelerated epigenetic age values (based on residuals to chronological age) for Levine’s age were significantly associated with higher reported BFI scores. |
| 52 | Rank= 1 | Robust rank aggregation method,  reported as a ranked value accounting for methylation measurements in multiple platforms. | The CpG site representing CD40 gene was the most differentially methylated position comparing cohorts of patients highly symptomatic patients experiencing psychiatric symptoms compared to those that do not in both EPIC and 450K illumine arrays. |
| 53 | Log Fold Change= 0.1057,  P= <0.05 | Limma regression,  reported as log-fold change | Beta values from CpG site cg03171795 for patients experiencing higher cumulative gastrointestinal symptoms were significantly different than those that did not. |
| 54 | Coefficient = −1.06,  P= 0.029 | Linear regression models,  reported as coefficient | Beta values from CpG site e cg14010619 were significantly associated with ototoxicity grade experienced. |
| 55 | Coefficient = 0.09  p= 1.52e-4 | Linear regression models,  reported as coefficient | Beta values from CpG site cg15417294 had significant associations with the occurrence of cardiomyopathy. |
| 57 | Coefficient= (-2.3)  P= <0.001 | Generalized estimating equation,  reported as coefficient | Higher accelerated epigenetic age values (based on residuals to chronological age) for Levine’s age were significantly associated with cumulative comorbidity score. |
